# Identifying Molecular Roadblocks for Transcription Factor-Induced Cellular Reprogramming In Vivo by Using *C. elegans* as a Model Organism

**DOI:** 10.3390/jdb11030037

**Published:** 2023-08-31

**Authors:** Ismail Özcan, Baris Tursun

**Affiliations:** Department of Biology, Institute of Cell and Systems Biology of Animals, University of Hamburg, 20146 Hamburg, Germany

**Keywords:** cellular reprogramming, transcription factor, reprogramming barrier, cell fate-safeguarding, *C. elegans*, RNAi

## Abstract

Generating specialized cell types via cellular transcription factor (TF)-mediated reprogramming has gained high interest in regenerative medicine due to its therapeutic potential to repair tissues and organs damaged by diseases or trauma. Organ dysfunction or improper tissue functioning might be restored by producing functional cells via direct reprogramming, also known as transdifferentiation. Regeneration by converting the identity of available cells in vivo to the desired cell fate could be a strategy for future cell replacement therapies. However, the generation of specific cell types via reprogramming is often restricted due to cell fate-safeguarding mechanisms that limit or even block the reprogramming of the starting cell type. Nevertheless, efficient reprogramming to generate homogeneous cell populations with the required cell type’s proper molecular and functional identity is critical. Incomplete reprogramming will lack therapeutic potential and can be detrimental as partially reprogrammed cells may acquire undesired properties and develop into tumors. Identifying and evaluating molecular barriers will improve reprogramming efficiency to reliably establish the target cell identity. In this review, we summarize how using the nematode *C. elegans* as an in vivo model organism identified molecular barriers of TF-mediated reprogramming. Notably, many identified molecular factors have a high degree of conservation and were subsequently shown to block TF-induced reprogramming of mammalian cells.

## 1. Introduction

**Figure 1:**
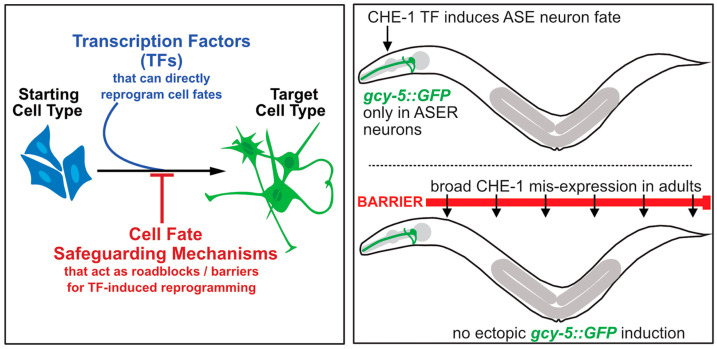
Illustration of the limitation during Transcription Factor-induced cell-fate reprogramming. Left: TF-induced direct reprogramming of cell identities is limited due to cell fate-safeguarding mechanisms that act as roadblock/barriers. Right: In *C. elegans.* The ZnF TF CHE-1 is required for terminal differentiation of the ASER and ASEL neurons in *C. elegans.* Only ASER is shown, which is labeled by the expression of the reporter transgene *gcy-5::gfp*. CHE-1 is normally expressed only in the two ASE neurons in the head of the animal. Broad overexpression of CHE-1 in all cells of the animals does not induce ectopic expression of the ASER reporter *gcy-5::gfp* in adult animals, indicating barriers exist to reprogram the cells.

Transcription factor (TF)-mediated reprogramming has the potential to deliver healthy cells to repair tissues and injured organs in patients. While the therapeutic potential of reprogrammed cells is evident, concerns regarding their safety remain [1]. One issue relates to whether specific cell types generated via reprogramming are fully converted, such that they will stay in a stably differentiated and functional state. Cell fate-safeguarding mechanisms limit or even block the reprogramming of the starting cell type [Figure 1] [2]. Hence, identifying and dissecting the molecular mechanisms of the diverse barriers is required to improve reprogramming efficiencies to establish a robust target cell identity. Model organisms such as the nematode *Caenorhabditis elegans* (*C. elegans*) are straightforward in vivo systems to identify molecular barriers of TF-mediated reprogramming by genetics and dissect the implicated molecular pathways.

Early observations of direct reprogramming or transdifferentiation events by using TF overexpression were described in *Drosophila melanogaster* and mouse embryonic fibroblasts (MEFs). In *Drosophila*, the forced overexpression of the homeodomain TF Antennapedia induced conversion of antennae to legs [3] and overexpression of paired homeobox TF eyeless induced ectopic eye structures instead of antennae [4]. Another landmark finding was the direct conversion of MEFs to muscle-like cells by the ectopic expression of the basic-Helix-Loop-Helix (bHLH) TF MyoD [5]. TF-mediated reprogramming climaxed with the finding that induced pluripotent stem cells (iPSCs) can be generated from differentiated cells such as mouse fibroblasts [6] and the subsequent demonstration of direct fibroblast reprogramming to a variety of different cell types, including neurons by overexpression of different TF-cocktails [7,8,9].

Yet, the abovementioned impediments of TF-induced cell-fate conversion caused by reprogramming barriers must be better understood. Several barrier factors for TF-induced reprogramming were identified in *C. elegans* and have a high degree of conservation. Notably, some were also shown to block TF-induced reprogramming in mammalian cells [10,11], indicating the power of *C. elegans*-based investigation of cellular reprogramming dynamics.

In this review, we summarize how using *C. elegans* has allowed the identification of molecular barriers that prevent TF-mediated reprogramming of germ and somatic cells in adult worms by using reverse genetic approaches (Table 1). We do not provide a comprehensive overview of all types of cellular reprogramming instances in *C. elegans,* such as developmentally programmed transdifferentiation [12,13] and other cases of cell-fate conversion phenomena, which were reviewed elsewhere recently [14,15].

## 2. Transcription Factor-Mediated Reprogramming of Cell Fates in *C. elegans*

The nematode *C. elegans* has a short generation time of around three days, during which it concludes embryonic development with hatching. After passaging through four larval stages (L1–L4), animals become fertile adult worms [22]. Developing cells of the early embryo display plasticity, and their lineages can be converted by overexpressing TFs without the need for depleting genes that may prevent lineage conversion [23,24]. Yet, embryonic cells progressively lose plasticity, in part due to the activity of epigenetic factors such as the repressive chromatin regulator Polycomb Repressive Complex 2 (PRC2) [25]. Accordingly, the 959 somatic cells of an adult hermaphrodite worm are terminally differentiated, while the germline keeps producing oocytes after sperm production is finalized during larval development. Notably, different molecular processes, such as chromatin regulation and translation control, protect germ cells against adverse somatic differentiation in order to maintain totipotency of the germline [26].

Studies in *C. elegans* demonstrated that the ectopic expression of various cell fate-inducing TFs can activate gene expression signatures that indicate a shift toward the target cell fate in the context of directly reprogramming one cell type into another [15]. One of the earliest studies in *C. elegans* showed that the ectopic expression of homeodomain (HD) type TF UNC-30 could induce the ectopic expression of genes specific to GABAergic motor neurons that regulate body locomotion in the animal [27]. In addition, the bHLH type TF HLH-1 (MyoD homolog) contributes to specifying the muscle fate and proper muscle functioning in *C. elegans* [28]. It was shown that forced overexpression of HLH-1 TF can reprogram non-muscle cell lineages in embryos into muscle-like cells [23,25]. Furthermore, studies in *C. elegans* showed that somatic lineages could be converted into endoderm by forced overexpression of single GATA-type TFs, such as END-1, END-3, or ELT-2, that regulate the specification and differentiation of endoderm [24,29]. Also, tissues change in response to ectopic expression of another GATA type TF ELT-7 and start resembling intestine-specific cellular morphologies [30,31]. Other examples of cell-fate reprogramming via inducing bHLH-type TFs include HLH-8 and LIN-32 TFs, which together specify the distal tip cell (DTC) identity in the male germline that is required to maintain the stem-cell niche in the germline of the animals. The forced overexpression of HLH-8 and LIN-32 together can reprogram the somatic anchor cells of the hermaphrodite vulva to male DTCs [32].

However, TFs that were expressed broadly in all tissues of an adult animal can induce ectopic fates in only a subset of cells, indicating the context dependency of successful reprogramming in vivo [33]. Conversion strategies of cells that are refractory to overexpressed TFs may require additional factors or the depletion of molecular reprogramming barriers [Figure 1]. Despite the progress in reprogramming, efficiency, heterogeneity, and context dependency remain significant challenges in TF-mediated cellular reprogramming. Studies focusing on the mechanisms that safeguard cell fate and counteract reprogramming in vivo improve our understanding of how cell fate is maintained and how to overcome barrier mechanisms in reprogramming.

## 3. Identifying Reprogramming Barriers in *C. elegans* Using Neuronal Fate Induction

The conversion of germ cells into neurons in *C. elegans* is a powerful in vivo reprogramming model for performing genetic screens to identify cellular safeguarding mechanisms. In *C. elegans*, the zinc finger type TF CHE-1 is required to terminally differentiate neurons to the identity of specific gustatory neurons known as ASER and ASEL [34,35]. The glutamatergic ASE neurons perceive chemosensory responses to navigate animals by taste and smell [34,36]. CHE-1 is exclusively expressed in ASER and ASEL and directly binds to cis-regulatory motifs in ASE-specific terminal differentiation genes encoding for signaling proteins, neurotransmitters, and others [34].

**Figure 2:**
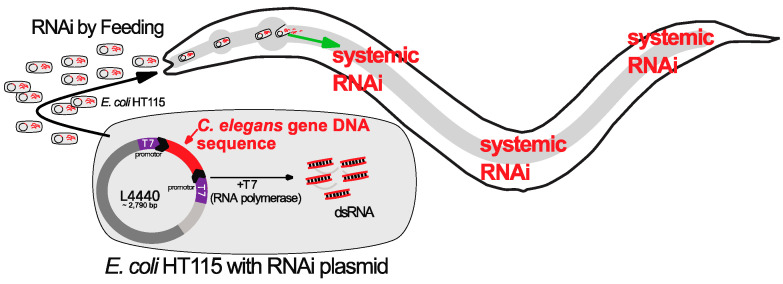
RNAi in *C. elegans*. *E. coli* bacteria (HT115) produce double stranded RNA (dsRNA) based on the plasmid L4440 with T7 promoters on both sides of the inserted target *C. elegans* gene. The ingested bacterial dsRNA trigger systemic RNAi.

**Figure 3:**
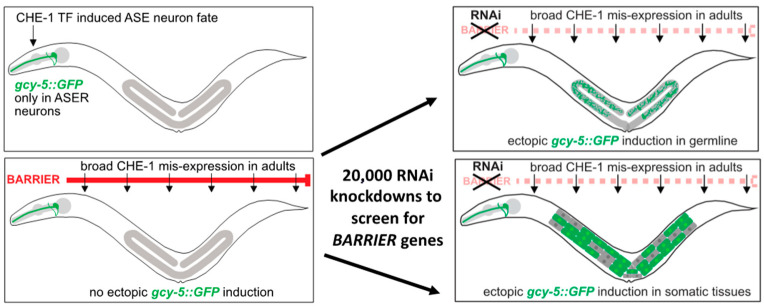
RNAi screening for reprogramming barriers. Using transgenic animals that overexpress the ZnF TF CHE-1 upon heat shock, a whole genome RNAi screen was conducted for animals that show ectopic expression of the ASER neuron reporter *gcy-5::gfp* in other tissues.

Broad overexpression of CHE-1 in most tissues of *C. elegans* has only limited capacity to induce ectopic expression of ASER-specific genes in other cells [16,37] [Figure 1]. The animals carry a transgene that allows heat shock-driven broad *che-1* overexpression and the ASER neuron fate reporter *gcy-5::GFP* reporter. The hypothesis that specific genes encode for factors that may act as barriers to restrict CHE-1 TF-induced conversion of cell identities prompted genetic screens by RNA interference (RNAi) [Figure 2] to identify reprogramming barriers [16,19,20,21]. Subsequent high-throughput RNAi screens have identified over 160 candidate genes that act as barriers for reprogramming of different cells into ASE neuron-like cells in *C. elegans* [16,19,20,21] [Figure 3]. The newly identified factors that block TF-induced cell-fate conversion are involved in diverse biological processes, including epigenetic regulation, protein regulation, and metabolism [16,19,20,21] (Table 1). Notably, identified factors often have conserved reprogramming roles and functions in mammalian cells, as demonstrated for the epigenetic factors LIN-53 and FACT [19,20,38,39].

## 4. RNAi Screens in *C. elegans* to Identify Reprogramming Barriers

In the laboratory of Oliver Hobert at Columbia University an RNAi screen was performed using transgenic *C. elegans* worms that allow overexpression of CHE-1 as described above [16]. The goal was to find genes that block CHE-1 TF-mediated direct reprogramming of cells to ASE neuron-like cells by RNAi-mediated knockdown of around 500 candidate genes with predicted functions in chromatin regulation [16]. RNAi can be applied in *C. elegans* by feeding worms with specific bacteria that generate RNA (dsRNA) from a plasmid complementary to the target *C. elegans* gene. The ingested bacterial dsRNA triggers systematic RNAi [40] [Figure 2]. It was found that RNAi against the histone chaperone LIN-53, in combination with broad CHE-1 overexpression, allowed the reprogramming of germ cells into ASE neuron-like cells [16].

Later, Kolundzic and colleagues extended the screening approach by performing a genome-wide RNAi screen to identify further genes that safeguard cell identities and counteract TF-mediated cell-fate reprogramming in vivo [19] [Figure 3]. This whole-genome screening approach identified approximately 160 target genes that allow ectopic induction of the ASER-neuron fate reporter *gcy-5::GFP* in various tissues such as the intestine, hypodermis, and germline upon depletion [19]. Gene ontology analysis (GO) of identified reprogramming factors from the genome-wide screen showed that they are involved in chromatin regulation, transcription, proteostasis, signaling, and mitochondrial processes [19]. Notably, amongst other factors, histone chaperone FACT (Facilitates Chromatin Transcription), which is an essential factor for stable gene expression by promoting transcription, was identified as a barrier for germ-cell reprogramming in *C. elegans* and will be described in more detail further below.

Furthermore, Hajduskova and colleagues established an automated RNAi screening strategy to identify more factors that prevent CHE-1-induced cell-fate reprogramming by generating an improved RNAi chromatin library to complement missing factors and correct false RNAi clones [20]. Using the updated chromatin RNAi library and automated RNAi screening pipeline, additional factors were identified to block CHE-1-mediated induction of ectopic ASER-neuron fate in various tissues [20].

The studies mentioned above suggested that cell-fate specification is often maintained and controlled by the activity of multiple genes in a context-dependent manner. Hence, investigating the loss of function of multiple genes via RNAi-mediated knockdown could provide additional reprogramming barriers. Simultaneous co-depletion of two genes via RNAi in *C. elegans* is usually performed by mixing two bacterial strains that contain specific dsRNA-producing plasmids each targeting an individual gene [41,42]. However, it has been shown that this approach often yields inefficient knockdown of both genes [43,44]. To overcome this issue, an improved method for robust double RNAi was established, which makes use of bacterial conjugation (**CONJU**gation-mediated **DO**uble **R**NAi technique = CONJUDOR) [21]. Double RNAi screening by CONJUDOR allowed the assessment of genes that are critical for animal developmental and viability in a large-scale manner in the context of TF-induced cell-fate reprogramming to GABAergic neuron-like cells [21]. Overexpression of the Pitx-type homeodomain TF UNC-30 specifying GABAergic motor neurons in *C. elegans* was used, which had been reported to induce the GABA fate marker *unc-25::GFP* in germ cells upon depletion of *lin-53* gene by RNAi [16]. However, the efficiency of *unc-25::GFP* induction in germ cells by overexpressed UNC-30 is limited in *lin-53* RNAi animals compared to *gcy-5::GFP* induction by CHE-1 [21]. Kazmierczak and colleagues reasoned that this difference might be due to additional barriers that prevent ectopic induction of the GABAergic motor neuron fate. Combining the *lin-53* RNAi clone with ∼800 other RNAi clones that target chromatin-related genes revealed that co-depletion of the Set1/MLL methyltransferase complex member RBBP-5 significantly increased the efficiency of germ-cell reprogramming to GABAergic neurons [21]. Hence, this study showed the importance of studying multiple genetic factors simultaneously to understand better cell fate-safeguarding mechanisms involved in TF-mediated direct reprogramming.

## 5. The Histone Chaperone LIN-53 Inhibits Germ Cell to Neuron Reprogramming

The histone chaperone LIN-53 was the first identified reprogramming factor that prevents direct reprogramming of germ cells into gustatory ASE neurons upon broad expression of ZnF-type TF CHE-1 [16]. While forced expression of CHE-1 alone in embryos resulted in ectopic expression of the ASE neuron fate marker *gcy-5::GFP*, broad CHE-1 misexpression in adult animals showed induced fate marker expression only in the head region of the animal, in particular only in sensory neurons [16]. Notably, the depletion of histone chaperone LIN-53 via RNAi-mediated knockdown in combination with overexpression of CHE-1 in adult worms led to the reprogramming of mitotic germ cells into ASE neuron-like cells [16]. The reprogrammed germ cells expressed pan-neuronal fate markers, including *rab-3*, *snb-1*, *unc-199*, *unc-33*, and *unc-10,* and also the specific neuron sub-type markers, such as *gcy-5* and *ceh-36*. In addition, reprogrammed germ cells lost their characteristic morphologies and acquired neuron-like cellular morphologies by developing axodendritic-like neuronal projections [16]. Moreover, these morphological changes were accompanied by the loss of germ-cell-specific perinuclear RNA granules termed P granules (also known as *C. elegans* germline granules), illustrating a faithful reprogramming of germ cells into neuron-like cells [16].

In addition to the ASE neuron fate, the depletion of histone chaperone LIN-53 permitted the reprogramming of the germ cells into other neuronal subtypes (Tursun et al., 2011). Forced expression of EBF-like TF UNC-3 or Pitx-type TF UNC-30 converted germ cells into cholinergic or GABAergic motor neurons, respectively [16]. Importantly, converted germ cells only expressed neuronal fate markers specific to the induced fate by the respective TF. For instance, in the CHE-1-reprogrammed germs cells, only ASE neuron-specific fate markers were expressed, suggesting that germ cells have not formed teratomas with mixed somatic cell types as known from other germ-cell conversion contexts [16,45]. Although the misexpression of the cell fate-inducing TFs (CHE-1, UNC-3, and UNC-30) and the RNAi-mediated depletion of LIN-53 were ubiquitous, neuronal induction occurred only in the germline [16]. These findings suggested that removing histone chaperone LIN-53 permits direct reprogramming into distinct neuronal subtypes in a germ line-specific manner.

Interestingly, the mammalian homolog of LIN-53, known as RBBP4/7 or CAF-1p48, is a core subunit of the CAF-1 histone chaperone complex and was later shown as a barrier for reprogramming of MEFs to neuron and induced pluripotent stem cells (iPSCs) [39]. This finding indicated that the role of LIN-53 as a reprogramming barrier is also conserved in mammalian cells.

## 6. LIN-53 Cooperates with Polycomb Repressive Complex 2 to Prevent Germ Cell to Neuron Reprogramming in *C. elegans*

The histone chaperone LIN-53 has diverse functions in chromatin biology as its part of many distinct multiprotein chromatin regulators, including NurD, CAF, HAT1, and Polycomb Repressive Complex 2 (PRC2) [17,46,47]. A follow-up study revealed that removing the components of the *C. elegans* PRC2 resulted in a similar outcome to that of the depletion of LIN-53 during the germ cell to neuron reprogramming [17]. PRC2 is an epigenetic regulator that represses chromatin via the deposition of H3K27 di- and trimethylation marks that are linked with developmentally regulated genes [48,49,50]. This finding was in line with the role of PRC2 in establishing repressive chromatin states in the germline genome [48,51]. Disruption of the repressive chromatin state in germ cells creates permissiveness to direct reprogramming into neurons [17,18]. Notably, this protective chromatin state might vary among different cell types since the loss of PRC2 only resulted in the induction of neuronal and muscle fate markers in germ cells but not in other somatic cell types (Patel et al., 2012).

PRC2-depletion-mediated reprogramming of germ cells to neurons is significantly enhanced in animals exhibiting high levels of Notch signaling activity via a gain-of-function mutation for the GLP-1 Notch receptor [52]. Germ-line-specific transcriptomic analysis demonstrated that elevated Notch signaling triggers the expression of PRC2-silenced genes, such as the expression of the H3K27 demethylase UTX1 [52]. Activation of UTX-1 antagonizes PRC2-mediated chromatin repression and results in an increased loss of silenced chromatin, leading to enhanced germ cell to neuron reprogramming [52]. Overall, these findings indicated that H3K27me3-mediated chromatin repression by PRC2 in collaboration with histone chaperone LIN-53 is an epigenetic state that inhibits TF-induced reprogramming of germ cells into neuron-like cells.

## 7. The FACT Complex Member HMG-3 Prevents TF-Mediated Germ Cell Reprogramming

FACT (FAcilitates Chromatin Transcription) is a heterodimeric histone chaperone complex that plays a crucial role in nucleosome remodeling by supporting RNA polymerase II during transcription [53,54,55]. In *C. elegans*, FACT consists of either one of the human SSRP1 orthologs HMG-3 or HMG-4 and the SUPT16H homolog SPT-16 [19]. All three *C. elegans* FACT components were identified as barriers to direct reprogramming of cells in *C. elegans*. FACT forms two tissue-specific isoforms in *C. elegans* composed of either HMG-3 or HMG-4 dimerized with SPT-16 [19]. HMG-3 is exclusively expressed in the germline, whereas HMG-4, which has approximately 90% amino acid similarity with HMG-3, is predominantly expressed in the soma, thereby forming germline and soma-specific FACT isoforms together with the ubiquitously expressed SPT-16 [19].

RNAi-mediated knockdown of *hmg-4* and *spt-16* genes led to the partial reprogramming of intestinal cells into neuron-like cells. In contrast, the depletion of *hmg-3* resulted in the reprogramming of germ cells into neuron-like cells [19]. Single-molecule fluorescence in situ hybridization (smFISH) confirmed that several neuron-specific genes were expressed in the reprogrammed germ and intestinal cells upon depletion of *hmg-3* or *hmg-4* and *spt-16*. However, the intestinal cells did not acquire neuron-like morphologies indicating partial reprogramming, probably due to additional biological constraints [19]. In contrast, HMG-3 depletion allowed the conversion of germ cells into ASE neuron-like cells with morphology features such as axodendritic projections and expression of multiple pan-neuronal and ASE neuron-specific marker genes, which were confirmed by applying smFISH to detect endogenous gene expression [19]. Notably, depletion of FACT alone without CHE-1 TF overexpression did not cause precautious induction of ectopic fates. Yet, the depletion of FACT subunits without inducing CHE-1 led to an impairment of cell fate maintenance of the germline and intestine, indicating that the permissiveness for reprogramming upon depletion of FACT is established by weakening the starting cell fate.

FACT is a highly conserved heterodimeric complex from worms to humans [19]. Kolundzic and colleagues demonstrated that FACT has a conserved reprogramming role and acts as a barrier for the reprogramming of human cells. Depletion of human FACT homologs SSRP1 and SUPTH16 using small interfering RNAs (siRNAs) enhanced TF-mediated reprogramming efficiency of human fibroblasts to iPSCs and neurons [19,56]. ATAC-seq and RNA-seq analysis suggested that loss of FACT increases ectopic chromatin accessibility and leads, among others, to decreased expression of factors known as reprogramming inhibitors, such as CAF-1 [39] or increased expression of reprogramming-promoting factors, such as SALL4 [57]. Overall, FACT was shown as an evolutionary conserved reprogramming barrier in *C. elegans* and human cells that protects cellular identity by maintaining gene expression profiles.

Interestingly, a FACT gene-related pseudogene termed *sspt-16* in *C. elegans* also acts as a TF-induced reprogramming barrier for germ cell to neuron conversion [58]. Lack of genomic *sspt-16* in a deletion mutant allows CHE-1-mediated reprogramming of germ cells to ASE neuron-like cells [58]. It is unclear how the deletion of the *sspt-16* locus causes permissiveness for germ-cell conversion. Deletion of the genomic *sspt-16* locus may cause loss of repressive chromatin, resulting in the spreading of active chromatin signatures and, thereby, allowing activation of ectopic gene expression.

## 8. The Chromodomain Protein MRG-1 Acts as a Reprogramming Barrier in the Germline

MRG-1 is a chromodomain protein and a component of the NuA4 histone acetyltransferase complex [59] and is an ortholog of the human MORF-related gene on chromosome 15 called MRG15 [60,61]. Previous studies have described that MRG-1 regulates the proliferation and differentiation of germ cells during development in *C.* elegans [62,63]. MRG-1 was recently identified as a reprogramming barrier during the reprogramming of germ cells into neurons upon forced expression of the ASE neuron fate-inducing TF CHE-1 [20]. The depletion of *mrg-1* using RNAi together with ectopic CHE-1 expression led to the reprogramming of germ cells into ASE neuron-like cells in *C. elegans*. Reporter gene expression, smFISH-based assessment of endogenous gene expression of neuronal genes, and loss of P-granules confirmed the faithful conversion of germ cells into neuron-like cells [20].

Further experiments have shown that, unlike the depletion of LIN-53 and PRC2, there were no changes in the levels of H3K27me3 in *mrg-1*-depleted animals [17,20]. This result indicated that MRG-1 acts as a reprogramming barrier via distinct mechanisms compared to LIN-53. In fact, germline versus soma-specific ChIP-seq analysis of genomic MRG-1 distribution showed that it primarily binds loci that possess the active chromatin marks H3K36me3, H3K9ac, and H3K4me3 [20]. This outcome suggested that MRG-1 is important to maintain germ cell identity-related gene expression profiles. Furthermore, immunoprecipitation of endogenous MRG-1 followed by mass-spectrometry (CoIP-MS) led to the identification of interacting proteins, including the ortholog of the mSin3A HDAC subunit SIN-3, the putative H3K9 methyltransferase SET-26, and the ortholog of the human O-GlcNAc transferase (OGT) OGT-1 [20]. Interestingly, animals carrying mutations for *sin-3*, *set-26*, or *ogt-1* synergistically enhanced the reprogramming efficiency upon depletion of *mrg-1* by RNAi, suggesting that these factors collaborate with MRG-1 in blocking the neuronal fate induction in the germline of *C. elegans* [20]. Extended CoIP-MS analysis identified another interactive partner of MRG-1: the Small Ubiquitin-like MOdifier (SUMO) [64,65]. MRG-1 undergoes post-translational modification by SUMO, which affects the chromatin binding pattern of MRG-1 [64]. Previous studies have indicated that SUMO also has functions in maintaining cell identity during the reprogramming of mammalian somatic cells [39,66]. Still, further investigation is needed to understand how SUMOylation of MRG-1 influences its function and whether it supports MRG-1 as a reprogramming barrier during germ cell to neuron reprogramming in *C. elegans*.

## 9. The Set1/MLL Methyltransferase Complex Member RBBP-5 Inhibits TF-Mediated Germ-Cell Reprogramming to GABAergic Neurons in *C. elegans*

Previous studies showed that the depletion of PRC2 subunits in *C. elegans,* including LIN-53, allowed the reprogramming of germs cells into neuron-like cells [16,17,52]. While RNAi against *lin-53* led to efficient germ-cell reprogramming into glutamatergic ASE neuron-like cells using overexpression of the ZnF TF CHE-1, reprogramming to GABAergic motor neurons by overexpressing the Pitx-type homeodomain TF UNC-30 was limited [21]. To improve germ-cell reprogramming by UNC-30, 700 chromatin factors were depleted together with LIN-53 by applying CONJUDOR. This led to identifying the Set1/MLL methyltransferase complex member RBBP-5 as a novel barrier for reprogramming germ cells into GABAergic motor neurons in *C. elegans* [21]. SET1/MLL is required to maintain H3K4 methylation in the germline [67]. Co-depletion of RBBP-5 and LIN-53 significantly enhanced the germ-cell reprogramming to GABAergic neurons. Yet, the mechanism of how LIN-53 and RBBP-5 may act in cooperation remains to be investigated.

## 10. Germ Granules (P granules) Safeguards Germ Cell Identity

Germ granules are perinuclear ribonucleoprotein aggregates present as membrane-less organelles from worm to human [26,68,69]. In *C. elegans*, loss of germ granules, also known as P granules, leads to sterility of worms [26,68,70]. Interestingly, RNAi against genes of P granule component-encoding genes, including *pgl-1*, *pgl-3*, *glh-1*, and *glh-4* leads to ectopic expression of somatic genes that are specific to neuronal and muscle fates [71,72]. This phenomenon does not require overexpression of a specific TF and is reminiscent of previously described teratoma-like formation in the *C. elegans* germline upon loss of translational regulators [45]. Yet, loss of P granules caused expression of pan-neuronal genes, but not those specific for neuron subtypes, suggesting that these neurons do not undergo terminal differentiation [72]. The added overexpression of the TF CHE-1, however, leads to expression of the ASER neuron fate reporter *gcy-5::gfp* suggesting that the germ cells gained sufficient permissiveness for extensive reprogramming upon loss of P granules [72]. Based on these findings, P granules can be considered as reprogramming barriers for TF-induced reprogramming in the germline; however, it should be considered that a teratoma-like state is created per se, which is generally undesired during the reprogramming of cells.

## 11. Summary and Future Perspective

Several factors have been identified as barriers for TF-induced reprogramming in *C. elegans* also by other genetic screens [16,19,20,21,33,52,58,73], suggesting that protection of cell fates is maintained via different molecular processes in living cells. As these barriers also appear to be conserved in mammalian cells [11], investigating the detailed molecular mechanisms by which these factors maintain cell fates may provide a better understanding of why some barriers act in a tissue or context-specific manner. This includes the role of germ granules, which are also considered as condensates [74] that may represent another layer of barriers for cellular reprogramming at least in the context of germ cells. Future studies combining single-cell transcriptome analysis with proteomics and metabolomics will provide more insight into how cell fates are safeguarded. Dissecting these mechanisms will improve the generation of homogeneous cell populations with high efficiency and increase safety aspects regarding the identity of the reprogrammed cells.

## Figures and Tables

**Table 1:** Overview of factors identified through genetic screens that block TF-mediated Direct Reprogramming in *C. elegans*.

Reprogramming Observed	Transcription Factor (TF)	Reprogramming Barrier	Function/Role	Human Counterpart	Reference
Germ cell to neuron	Znf TF CHE-1 ZnF TF UNC-3 Homeobox UNC-30	LIN-53	Histone Chaperone	RBBP4/7	Tursun et al., 2011 [16]
Germ cell to neuron	ZnF TF CHE-1	PRC-2 (MES-2/-3)	H3K27 Methylation	EZH2	Patel et al., 2012 [17]
Germ cell to muscle	bHLH TF HLH-1	PRC-2 (MES-6)	H3K27 Methylation	EED, MyoD, MYF5,6	Patel et al., 2012 [17]
Germ cell to neuron	ZnF TF CHE-1	MES-4	Histone Chaperone H3K36 Methylation	NSD proteins	Gaydos et al., 2012 [18]
Germ cell and intestine to neuron	ZnF TF CHE-1	FACT (HMG-3/-4, SPT-16)	Histone Chaperone	SSRP1, SUPT16H	Kolundzic et al., 2018 [19]
Germ cell to neuron	ZnF TF CHE-1	MRG-1	Part of NuA4 histone acetyltransferase complex	MRG-15	Hadjuskova et al., 2019 [20]
Germ cell to neuron	ZnF TF CHE-1 Homeobox UNC-30	RBBP-5	Set1/MLL methyltransferase complex member	RBBP5	Kazmierczak et al., 2021 [21]
